# Supplementary material for: Reconstitution of human DNA licensing and the structural and functional analysis of key intermediates
Source: Nat Commun. 2025 Jan 8;16:478. doi: 10.1038/s41467-024-55772-z (PMC11711466; doi:10.1038/s41467-024-55772-z)
Supplement: Supplementary file 1 — Supplementary Information [file 41467_2024_55772_MOESM1_ESM.pdf]

SUPPLEMENTARY INFORMATION

Reconstitution of human DNA licensing and the structural and functional analysis of key intermediates

Jennifer N. Wells[1,2,*], Lucy V. Edwardes[1,2*], Vera Leber[1,2] , Shenaz Allyjaun[1,2], Matthew Peach[1,2], Joshua Tomkins[1,2], Antonia Kefala-Stavridi[1,2], Sarah V. Faull[1,2], Ricardo Aramayo[1,2], Carolina M. Pestana[1,2], Lepakshi Ranjha[1,2] and Christian Speck[1,2]

[1] DNA Replication Group, Institute of Clinical Sciences, Faculty of Medicine, Imperial College London, London W12 0HS, UK

[2] MRC Laboratory of Medical Sciences (LMS), W12 0HS London, United Kingdom.

[*] These authors contributed equally

Correspondence: chris.speck@imperial.ac.uk

This file contains Supplementary Figures 1 to 10 and Supplementary Table 1.

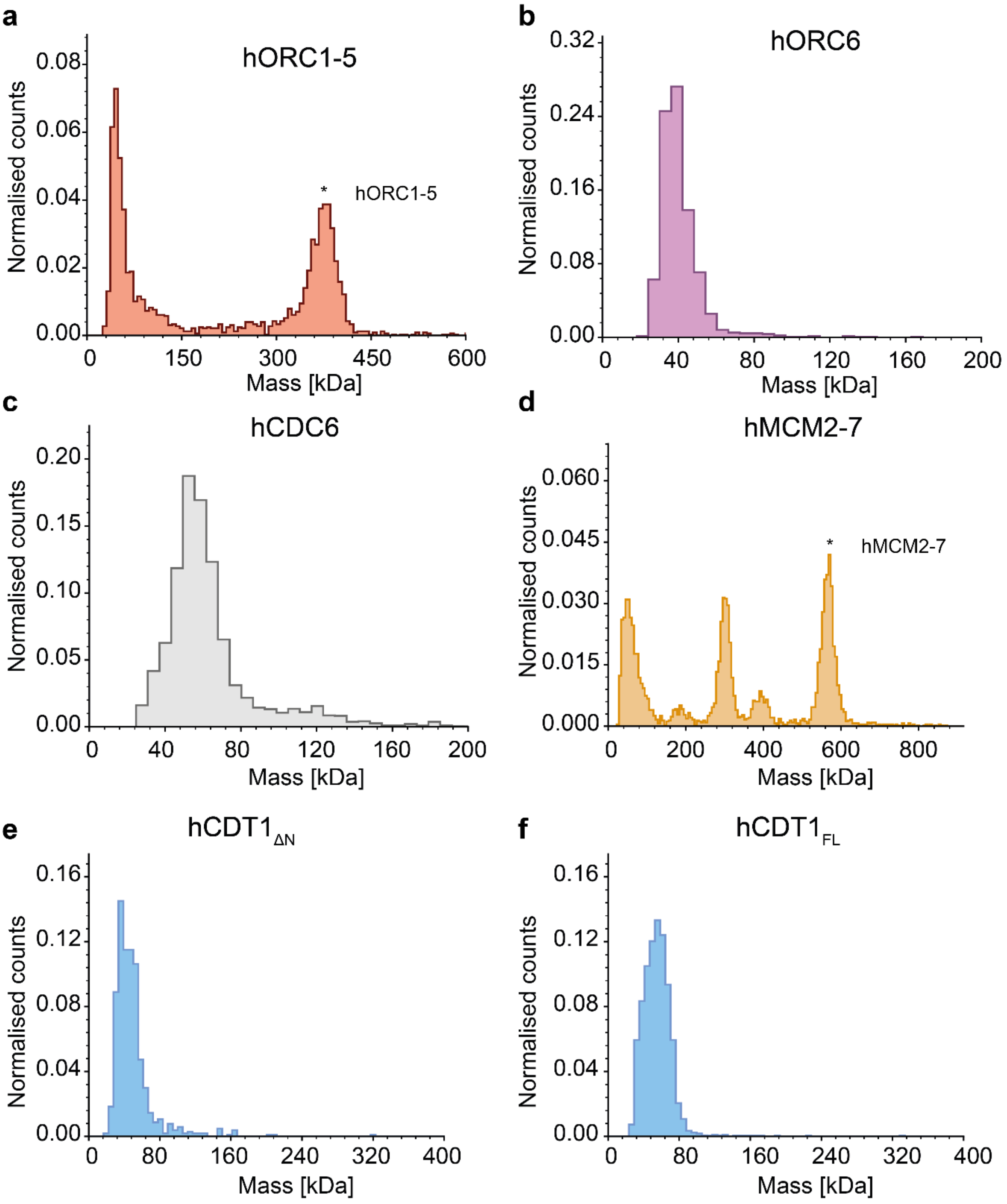

Supplementary Fig. 1: **Mass photometry histograms of purified proteins used in the pre-RC assay**. (**a**) hORC1-5, (**b**) hORC6, (**c**) hCDC6, (**d**) hMCM2-7 (**e**) hCDT1$_{\Delta N}$ truncation mutant and (**f**) hCDT1$_{FL}$. We observed multiple masses for hMCM2-7 due to limited complex stability at low concentrations of protein used for the mass photometry analysis. Note that high protein concentrations are not amenadable for mass photometry analysis. Source data are provided as a Source Data file.

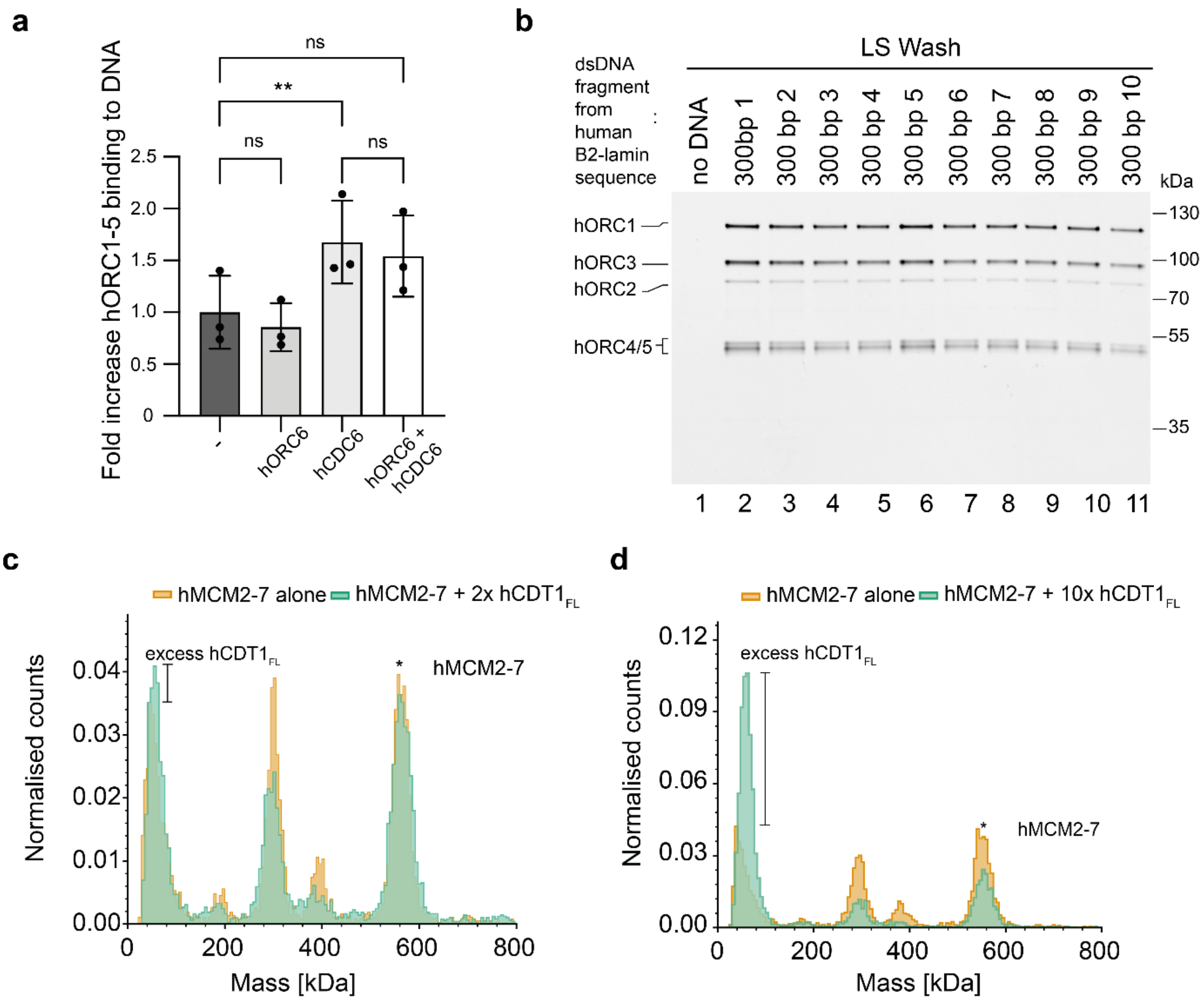

Supplementary Fig. 2: **DNA sequence analysis of DNA licensing and interaction analysis of hCDT1-hMCM2-7**. (**a**) Fold enhancement of hORC1-5 signal after low salt wash conditions in the absence (-) or presence of hORC6, hCDC6 or both hORC6 and hCDC6 (Lane 1-4 from Fig. 1c). Mean plotted; individual data points are marked with black circles. n=3 independent experiments, error bars represent standard deviation, statistical significance was calculated using RM one-way ANOVA with Tukey's multiple comparisons test, ns – not significant, **P≤0.01, P=0.0078. (**b**) Pre-RC like assay, hORC1-5 was incubated with 300 bp dsDNA fragments along the length of the human B2-lamin sequence and washed under low salt wash conditions. SDS-PAGE gel is representative of three independent biological replicates. (**c**) In solution complex formation was not observed between hMCM2-7 and $hCDT1_{FL}$ when $hCDT1_{FL}$ was used in 2xfold excess over hMCM2-7. (**d**) A 10fold excess of $hCDT1_{FL}$ over hMCM2-7 also failed to induce hCDT1 interaction with hMCM2-7. (c-d) Mass photometry is carried out in the presence of ATP. hMCM2-7 peaks are marked with an asterisk. Source data are provided as a Source Data file.

a

Sample Preparation

- → in-solution pre-RC assay +/- hORC6
- → prepare NS grids after:
  - ↳ 20 min and 60 min
- → repeat n=3

Data Collection

Collect ≥ 400 Micrographs per experiment

Data Processing: CryoSPARC

- → Import Micrographs (Mics.)
- → Blob Picker
- → Extract Particles
- → 2D Classification
- → Subset Selection
  - ↳ Remove junk
- → 2D Classification
- → Subset Selection
  - ↳ Cleaned particle subset (100%)
- → Subset Selection
  - ↳ Unambiguous Double Hexamers (DH)

b

+ ORC6	Mics.	Extract	Clean	DH	DH 2D Class Averages	% DH
20 min						
n1)	404	258772	179111	12114	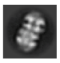	7%
n2)	450	225251	88286	3084	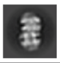	3%
n3)	400	177492	78877	9577	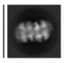	12%
			+ORC6, 20 min, Mean			7%
			+ORC6, 20 min, Standard deviation			4%
60 min						
n1)	408	112803	78318	15824	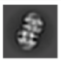	20%
n2)	441	183689	72301	7724	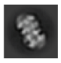	11%
n3)	400	157304	86719	10983	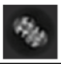	13%
			+ORC6, 60 min, Mean			15%
			+ORC6, 60 min, Standard deviation			4%
- ORC6						
20 min						
n1)	480	258993	66658	4977	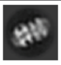	7%
n2)	483	174111	59939	1786	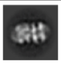	3%
n3)	480	189226	85118	2817	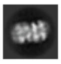	3%
			-ORC6, 20 min, Mean			4%
			-ORC6, 20 min, Standard deviation			2%
60 min						
n1)	408	117758	65525	0		0%
n2)	471	131171	73702	2656	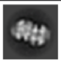	4%
n3)	400	173221	72786	2666	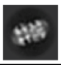	4%
			-ORC6, 60 min, Mean			3%
			-ORC6, 60 min, Standard deviation			2%

Supplementary Fig. 3: **Negative stain EM data analysis for incidence of hMCM2-7 double hexamers using in-solution DNA licensing assay.** (**a**) Overview of sample preparation, data collection, and data processing workflow and logic. (**b**) Image processing and statistics from all data collections displaying number of repeats (n), number of micrographs collected (Mics.), total number of particles extracted after particle picking with gausian blob (Extract), the total number of particles after two rounds of 2D classification. Ambiguous, low resolution particle classes and aggregates were excluded to ensure unambiguous reporting of double hexamers in context of a heterogeneous in-solution assay containing individual licensing factors and various intermediate complexes (Clean), and finally, the number of particles within the 2D classes corresponding to double hexamers (DH). Class averages corresponding of selected double hexamers are displayed (DH 2D class averages). The percent of double hexamers was calculated as the ratio between the number of double hexamer particles to the number of clean particles. Standard deviations were calculated based on three experimental repeats.

Supplementary Table 1 Summary of Cryo-EM Data Collection and Model Refinement

	hOCCM
**Data Collection/Processing**	
Voltage (kV)	300
Magnification	81,000
Defocus range (µm)	-0.5 to -2.1 (0.2)
Symmetry imposed	C1
Total electron dose (e-/ A^2)	40
Exposure Time (s)	3.0
Number of micrographs	18,728
Number of frames/micrograph	40
Initial Particle Number	334,603
Final Particle Number	8,730
Resolution (masked, Å)	6.09
FSC threshold	0.143
**Refinement**	
Model composition	
Protein Residues	6369
Nucleotides	79
Ligand	0
RMS Deviations	
Bond Lengths (Å)	0.24
Bond Angels (degree)	0.49
Ramachandran	
Favoured (%)	87
Allowed (%)	12
Outlier (%)	2
MolProbity Score	5.67

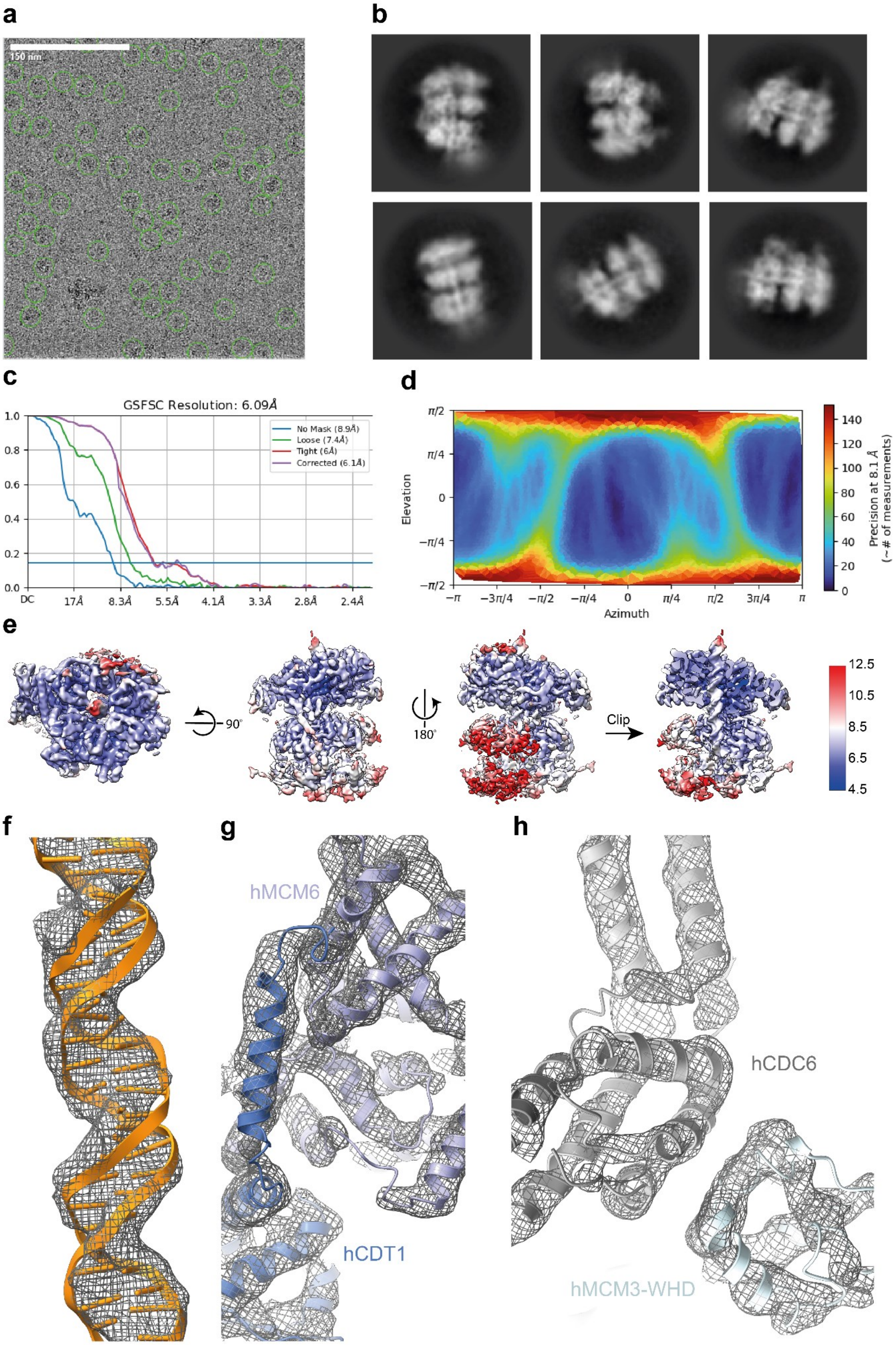

Supplementary Fig. 4: Quality of the EM data. (**a**) A representative micrograph from the hOCCM dataset was collected on a Titan Krios TEM operated at 300 kV and a K3 direct electron detector. Particle coordinates are circled in green. Scale bar is 150 nm. (**b**) 2D class averages show the hOCCM

complex bound to DNA. (**c**) CryoSPARC FSC curve from final 3D model refinement indicating 6.09 Å resolution with 0.143 cut-off. (**d**) Angular distribution of the particles included in final 3D map. (**e**) Local resolution representation of the complex. (**f**) Experimental density map showing fit of dsDNA (orange) model. (**g**) Experimental density map showing local fit between hCDT1 interacting with hMCM6 in the hOCCM model. (**h**) Experimental density map showing model fit for the described hCDC6-hMCM3 interaction within the hOCCM structure.

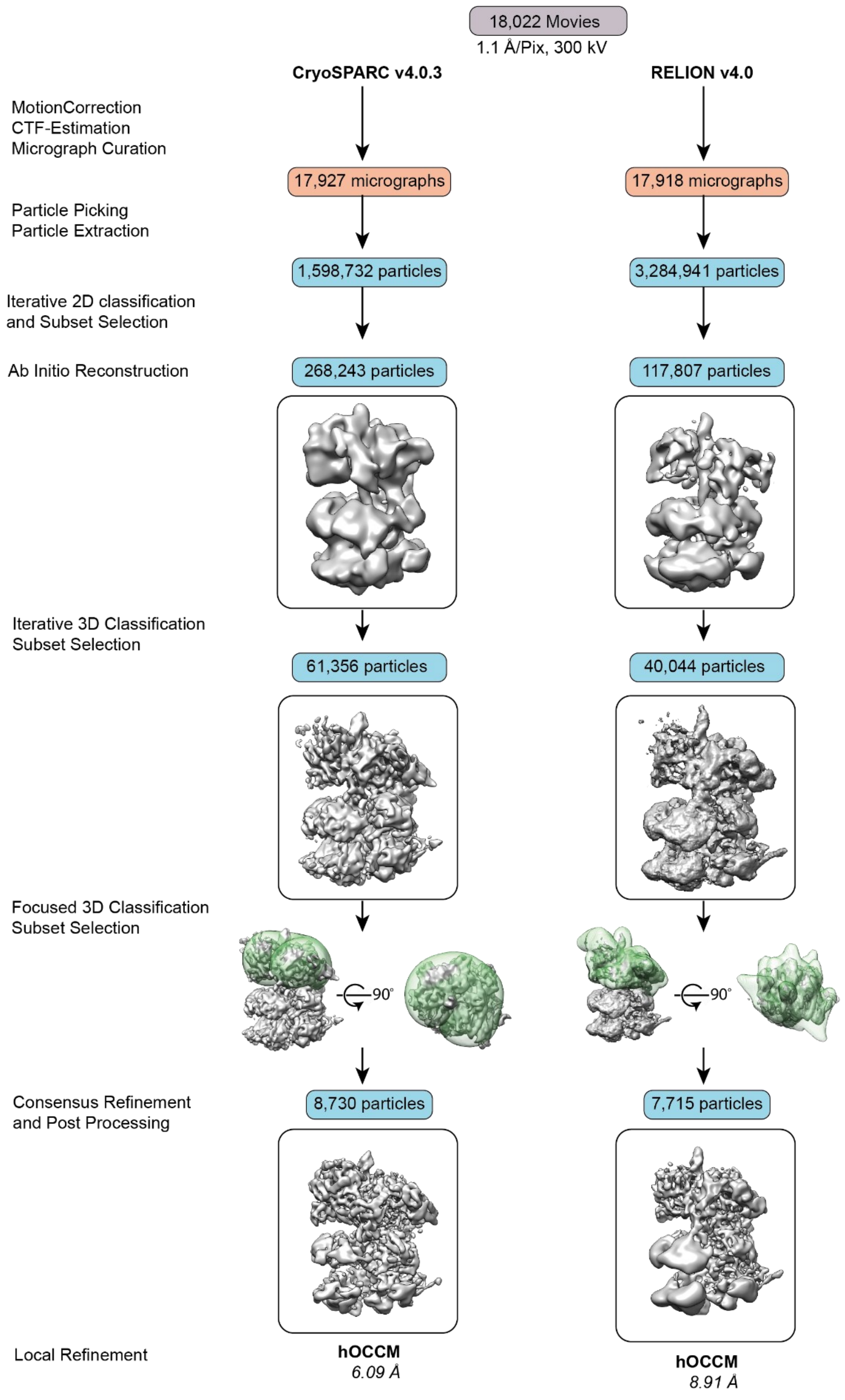

Supplementary Fig. 5: Image processing workflow. The hOCCM structure could be resolved by carrying out image processing in both cryoSPARC v4.03 (left) or Relion v4.0 (right). Due to orientation bias and local flexibility, both software's resolved a final consensus structure with less than 10,000 particles in the final particle subset.

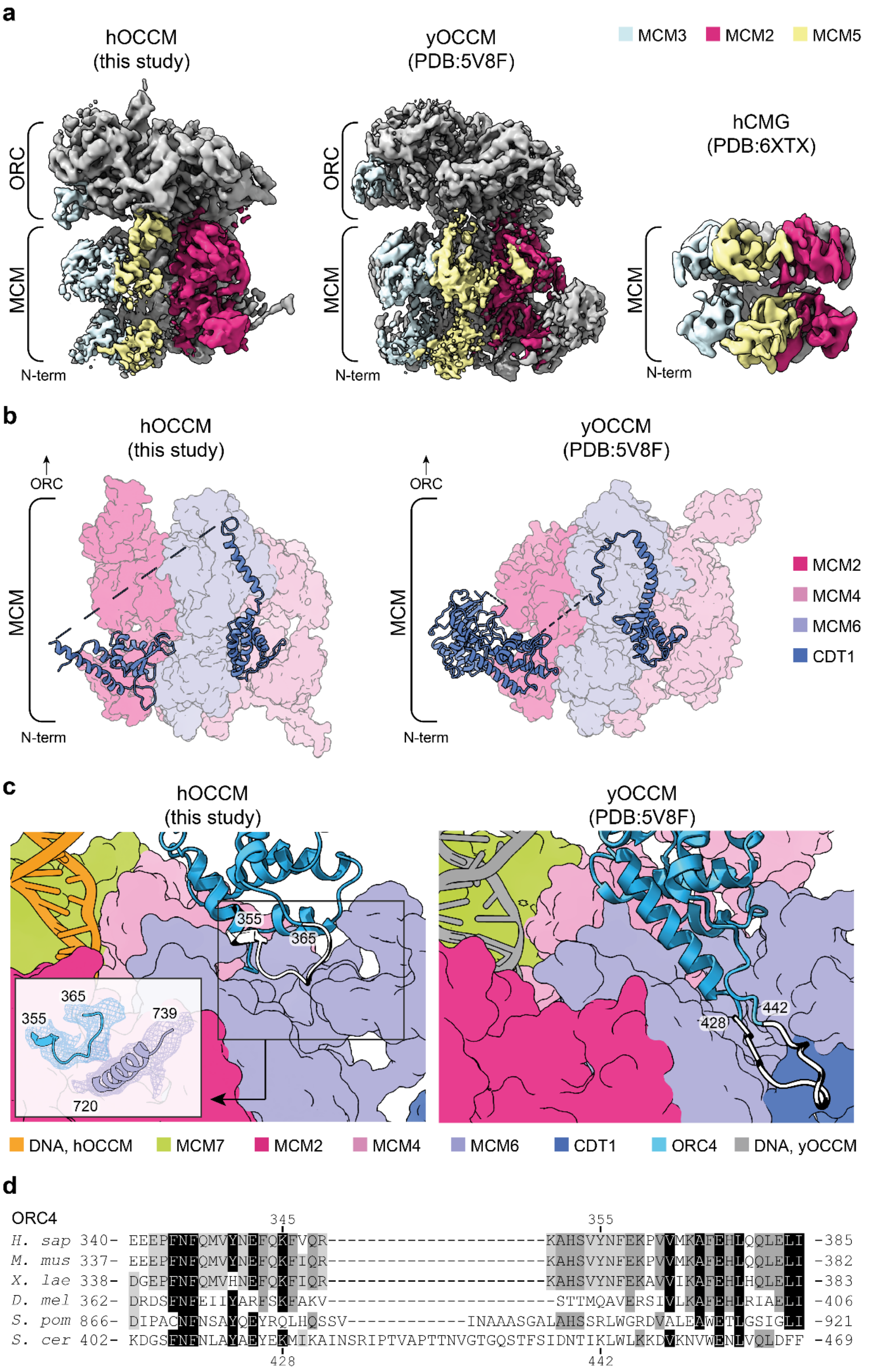

Supplementary Figure 6: Comparisons between the hOCCM and yOCCM. (**a**) hOCCM map (left) showing the flexibility of hMCM5 at the 2/5 gate compared to the experimental map for the yOCCM (PDB: 5V8f, centre) and experimental density for the hCMG (PDB: 6XTX, right). All maps are displayed at similar contour levels and have been coloured to highlight experimental density corresponding to MCM2 (dark pink), MCM3 (blue), and MCM5 (yellow). (**b**) Molecular models depicting conservation of

the binding position for CDT1 (blue) bound to MCM2 (dark pink) MCM6 (purple) and MCM4 (light pink), with MCM2-7 complexes displayed as surfaces in the hOCCM (left) and yOCCM (PDB: 5V8F, right). (**c**) ORC4 loop position relative to hMCM6 WHD in hOCCM compared to yOCCM, with inset showing map to model fits for this region of hORC4 and hMCM6. (**d**) Protein sequence alignment for ORC4 loop region.

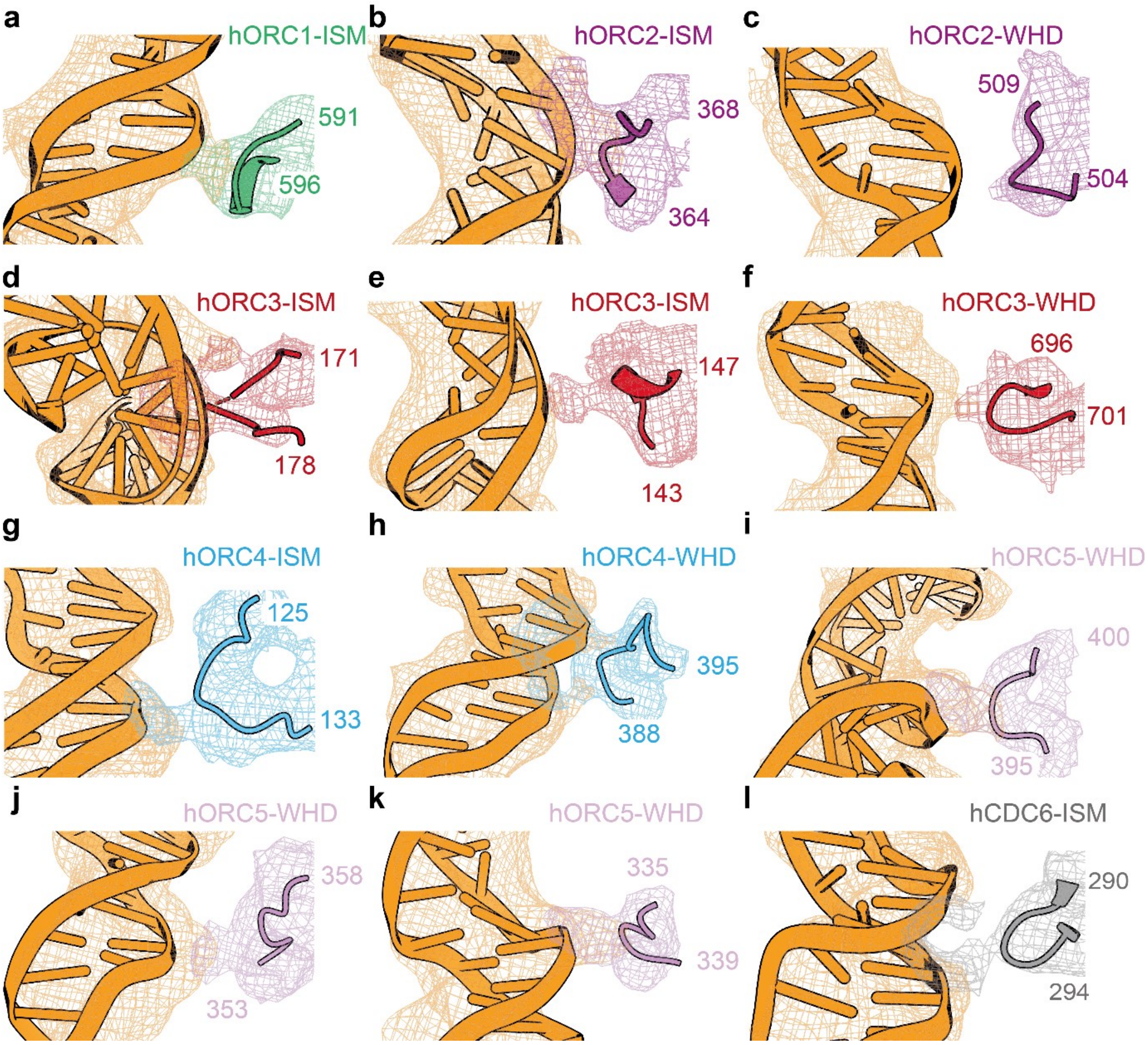

Supplementary Fig. 7: Local experimental densities corresponding to Fig 8b. Map to model fits showing dsDNA local to regions of the (**a**) hORC1-ISM, (**b**) hORC2-ISM and (**c**) hORC2-WHD, (**d-e**) the hORC3-ISM and (**f**) hORC3-WHD, (**g**) the hORC4-ISM and (**h**) hORC4-WHD, (**i-k**) the hORC5-WHD and finally, (**l**) the hCDC6-ISM region spanning in the hOCCM structure.

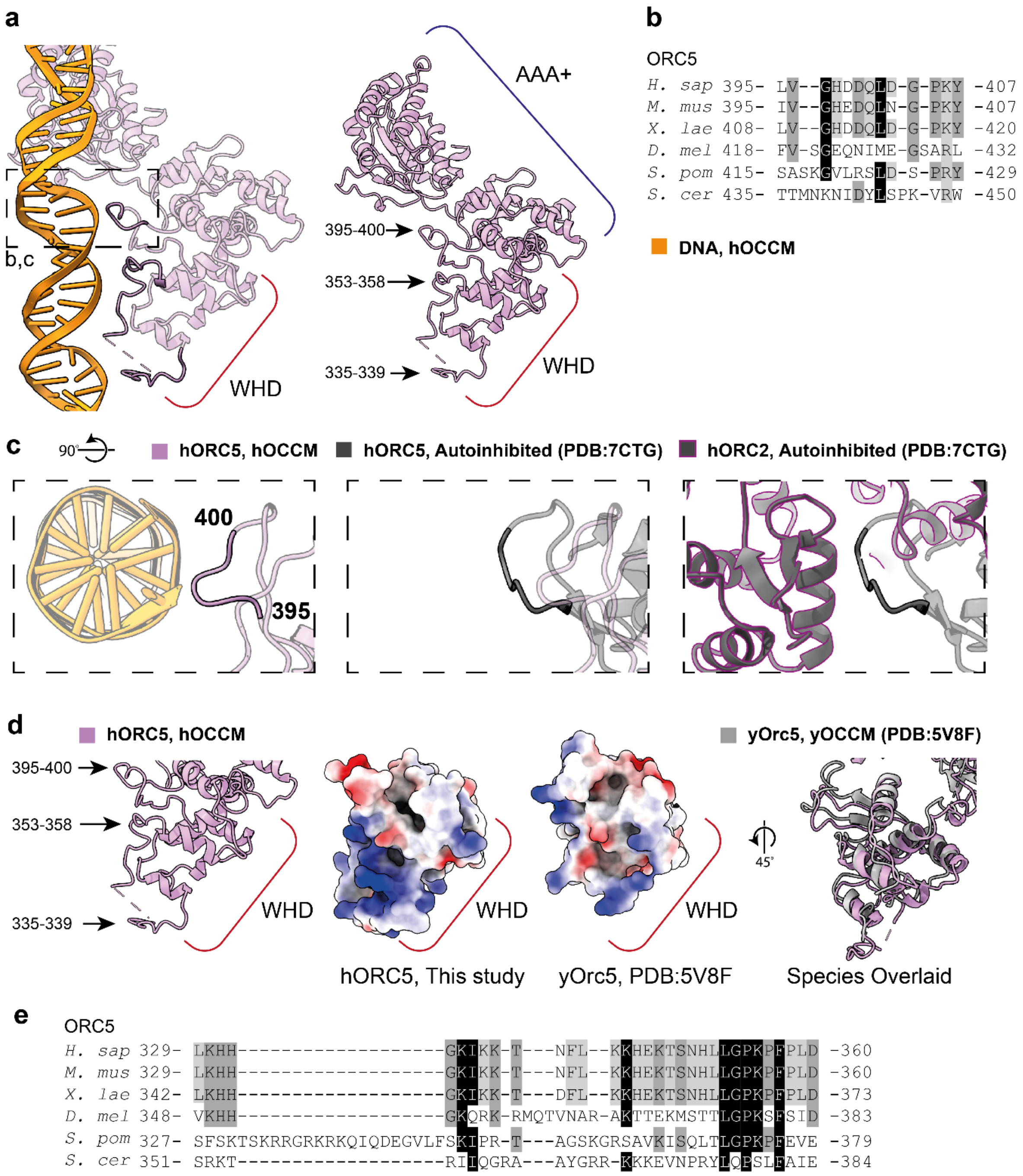

Supplementary Fig. 8: hORC5 makes multiple DNA backbone contacts. (**a**) The hORC5-WHD makes three contacts with the phosphate backbone. (**b**) Sequence alignment of the hORC5 aa395-400 DNA contacts. (**c**) hORC5 aa395-400 makes contact with DNA, but in the autoinhibited state, the same region interacts with hORC2. (**d**) Comparison of the hORC5-WHD and the yeast counterpart. Electrostatic potential charge surface representation is shown (blue represents positive charge, white neutral charge and red negative charge). (**e**) Sequence alignment of hORC5 aa329-360, highlighting a conserved sequence in higher eukaryotes.

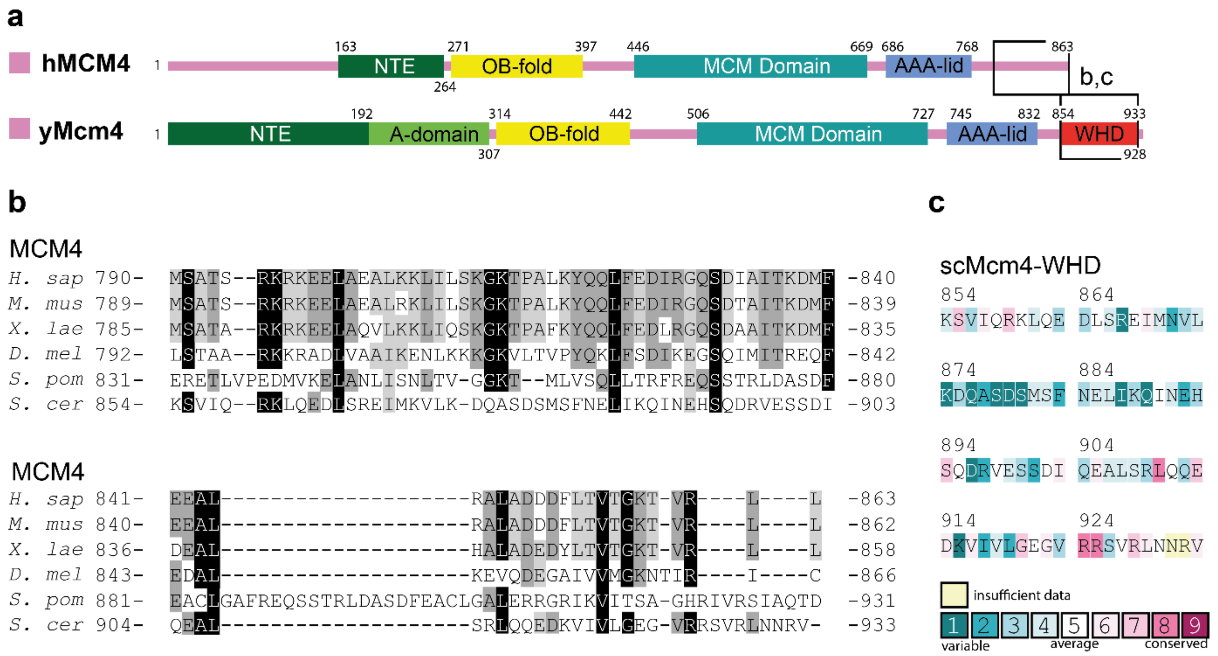

Supplementary Fig. 9: C-terminal regions of Mcm4 diverge. (**a**) Side-by-side comparison of the domain organisation of MCM4 in human (top) and yeast (bottom). (**b**) Alignment of the C-terminus of MCM4 highlighting its divergence during evolution. (**c**) Representation of the yMcm4-WHD coloured by conservation as per the ConSURF database[1] using chain 4 of the yOCCM (PDB:58VF).

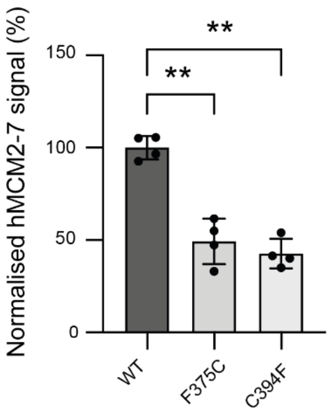

Supplementary Fig. 10: Cosmic mutations in hCDC6 impact high salt-stable hMCM2-7 loading. Normalised hMCM2-7 signal with $hCDC6_{WT}$ and two hCDC6 cosmic mutants (F375C and C394F) after high salt wash conditions (lane 4-6 from Fig. 10e). Mean plotted; individual data points are marked with black circles. Mean plotted; individual data points are marked with black circles. n=4 independent experiments, error bars represent standard deviation, statistical significance was calculated using RM one-way ANOVA with Tukey's multiple comparisons test, **P≤0.01, P = 0.0012 WT vs F375C and P = 0.0060 WT vs C394F). Source data are provided as a Source Data file.

Reference:

1. Yariv, B. et al. Using evolutionary data to make sense of macromolecules with a "face-lifted" ConSurf. *Protein Sci* **32**, e4582 (2023).
