# Supplementary material for: Reconstitution of human DNA licensing and the structural and functional analysis of key intermediates
Source: Nat Commun. 2025 Jan 8;16:478. doi: 10.1038/s41467-024-55772-z (PMC11711466; doi:10.1038/s41467-024-55772-z)
Supplement: Supplementary file 2 — Description of Additional Supplementary Files [file 41467_2024_55772_MOESM2_ESM.pdf]

Description of Additional Supplementary Files

Supplementary Data 1: Sequences of oligonucleotides and a list of all plasmids mentioned in this study
