# Supplementary material for: Reconstitution of human DNA licensing and the structural and functional analysis of key intermediates
Source: Nat Commun. 2025 Jan 8;16:478. doi: 10.1038/s41467-024-55772-z (PMC11711466; doi:10.1038/s41467-024-55772-z)
Supplement: Supplementary file 4 — Reporting Summary [file 41467_2024_55772_MOESM4_ESM.pdf]

nature portfolio

Corresponding author(s): Christian Speck

Last updated by author(s): Dec 6, 2024

Reporting Summary

Nature Portfolio wishes to improve the reproducibility of the work that we publish. This form provides structure for consistency and transparency in reporting. For further information on Nature Portfolio policies, see our Editorial Policies and the Editorial Policy Checklist.

Statistics

For all statistical analyses, confirm that the following items are present in the figure legend, table legend, main text, or Methods section.

n/a	Confirmed	
☐	☒	The exact sample size (*n*) for each experimental group/condition, given as a discrete number and unit of measurement
☐	☒	A statement on whether measurements were taken from distinct samples or whether the same sample was measured repeatedly
☐	☒	The statistical test(s) used AND whether they are one- or two-sided<br>*Only common tests should be described solely by name; describe more complex techniques in the Methods section.*
☐	☒	A description of all covariates tested
☐	☒	A description of any assumptions or corrections, such as tests of normality and adjustment for multiple comparisons
☐	☒	A full description of the statistical parameters including central tendency (e.g. means) or other basic estimates (e.g. regression coefficient) AND variation (e.g. standard deviation) or associated estimates of uncertainty (e.g. confidence intervals)
☐	☒	For null hypothesis testing, the test statistic (e.g. *F*, *t*, *r*) with confidence intervals, effect sizes, degrees of freedom and *P* value noted<br>*Give P values as exact values whenever suitable.*
☒	☐	For Bayesian analysis, information on the choice of priors and Markov chain Monte Carlo settings
☒	☐	For hierarchical and complex designs, identification of the appropriate level for tests and full reporting of outcomes
☒	☐	Estimates of effect sizes (e.g. Cohen's *d*, Pearson's *r*), indicating how they were calculated

Our web collection on statistics for biologists contains articles on many of the points above.

Software and code

Policy information about availability of computer code

Data collection	Data Collection: Gatan Digital Micrograph, TEM UI v2.5.1(Talos) , ThermoFischer EPU (Krios), v2.9.0.1519REL (Talos), AcquireMP v2023R2
Data analysis	cryoSPARC v4.03, UCSF Chimera v1.16, ChimeraX-1.3, COOT v0.9.7, Phenix v10.20.1, Multi Gauge v2.3, GraphPadPrism v10.2, DiscoverMP v2023R2, Relion v4.0

For manuscripts utilizing custom algorithms or software that are central to the research but not yet described in published literature, software must be made available to editors and reviewers. We strongly encourage code deposition in a community repository (e.g. GitHub). See the Nature Portfolio guidelines for submitting code & software for further information.

Data

Policy information about availability of data

All manuscripts must include a data availability statement. This statement should provide the following information, where applicable:

- Accession codes, unique identifiers, or web links for publicly available datasets
- A description of any restrictions on data availability
- For clinical datasets or third party data, please ensure that the statement adheres to our policy

The EM map and associated atomic model of the hOCCM reported in this study have been deposited to the Electron Microscopy Data Bank (EMDB) and the RCSB Protein Data Bank (PDB) under accession numbers EMD-19566/PDB:8RWV, respectively. Other data that support the study are available from the corresponding author upon reasonable request.

Research involving human participants, their data, or biological material

Policy information about studies with human participants or human data. See also policy information about sex, gender (identity/presentation), and sexual orientation and race, ethnicity and racism.

Reporting on sex and gender	N/A
Reporting on race, ethnicity, or other socially relevant groupings	N/A
Population characteristics	N/A
Recruitment	N/A
Ethics oversight	N/A

Note that full information on the approval of the study protocol must also be provided in the manuscript.

Field-specific reporting

Please select the one below that is the best fit for your research. If you are not sure, read the appropriate sections before making your selection.

☒ Life sciences ☐ Behavioural & social sciences ☐ Ecological, evolutionary & environmental sciences

For a reference copy of the document with all sections, see nature.com/documents/nr-reporting-summary-flat.pdf

Life sciences study design

All studies must disclose on these points even when the disclosure is negative.

Sample size	No statistical methods were used to predetermine sample size, as much data as possible was collected during allocated instrument session time. We collected 334603 particles from 18022 micrographs for hOCCM.
Data exclusions	Data was excluded at different stages of image processing. Particles that did not meet the image resolution quality criteria or could not be averaged were excluded from the final structures, based on 2D averages and 3D map classification results. The method of data exclusion is common practice in the cryo-EM field to allow for the generation of interpretable 3D models.
Replication	Biochemical experiments were repeated at least three times as biological replicates.
Randomization	Experiments were not randomized due to the nature of the data not involving patients or animals. The experiments were not grouped and needed to be preformed in a particular order.
Blinding	Blinding is not applicable to this research, which does not entail randomization and does not involve patients or animals. The experiments required essential visual inspection of the data quality during data screening and data collection and so no blinding could be performed.

Reporting for specific materials, systems and methods

We require information from authors about some types of materials, experimental systems and methods used in many studies. Here, indicate whether each material, system or method listed is relevant to your study. If you are not sure if a list item applies to your research, read the appropriate section before selecting a response.

Materials & experimental systems

n/a	Involved in the study	
☐	☒	Antibodies
☐	☒	Eukaryotic cell lines
☒	☐	Palaeontology and archaeology
☒	☐	Animals and other organisms
☒	☐	Clinical data
☒	☐	Dual use research of concern
☒	☐	Plants

Methods

n/a	Involved in the study	
☒	☐	ChIP-seq
☒	☐	Flow cytometry
☒	☐	MRI-based neuroimaging

Antibodies

Antibodies used	Anti-hCdc6 mouse monoclonal antibody (1:1,000), Santa Cruz, Cat# sc-9964<br>Anti-hCdt1 mouse monoclonal antibody (1:1,000), Santa Cruz, Cat# sc-365305

Anti-Mouse HRP-linked goat polyclonal antibody (1:10,000), Sigma, Cat# A4416

Validation	In this study, antibodies to human DNA replication proteins were used in Western blots to probe factor retention under variable conditions in the pre-RC assay. The manufacturer performed a Western blot analysis of endogenous proteins from whole cell lysates. In addition, we could verify these results independently using whole cell lysates isolated from RPE-1 hTert cells and purified proteins .

Eukaryotic cell lines

Policy information about cell lines and Sex and Gender in Research

Cell line source(s)	HEK293 cells were used for overexpression of the hMCM2-7 complex, as reported previously in Rzechorzek, et.a., (2020), and were provided by Oxford expression technologies LTD.
Authentication	Cell line was not authenticated.
Mycoplasma contamination	Cell line was negative for mycoplasma.
Commonly misidentified lines (See ICLAC register)	*Name any commonly misidentified cell lines used in the study and provide a rationale for their use.*

Plants

Seed stocks	*Report on the source of all seed stocks or other plant material used. If applicable, state the seed stock centre and catalogue number. If plant specimens were collected from the field, describe the collection location, date and sampling procedures.*
Novel plant genotypes	*Describe the methods by which all novel plant genotypes were produced. This includes those generated by transgenic approaches, gene editing, chemical/radiation-based mutagenesis and hybridization. For transgenic lines, describe the transformation method, the number of independent lines analyzed and the generation upon which experiments were performed. For gene-edited lines, describe the editor used, the endogenous sequence targeted for editing, the targeting guide RNA sequence (if applicable) and how the editor was applied.*
Authentication	*Describe any authentication procedures for each seed stock used or novel genotype generated. Describe any experiments used to assess the effect of a mutation and, where applicable, how potential secondary effects (e.g. second site T-DNA insertions, mosiacism, off-target gene editing) were examined.*
